# Characterization of Metabolite Landscape Distinguishes Medicinal Fungus *Cordyceps sinensis* and other *Cordyceps* by UHPLC-Q Exactive HF-X Untargeted Metabolomics

**DOI:** 10.3390/molecules28237745

**Published:** 2023-11-24

**Authors:** Chuyu Tang, Xiuzhang Li, Tao Wang, Jie Wang, Mengjun Xiao, Min He, Xiyun Chang, Yuejun Fan, Yuling Li

**Affiliations:** 1 State Key Laboratory of Plateau Ecology and Agriculture, Qinghai Academy of Animal and Veterinary Sciences, Qinghai University, Xining 810016, China; chuyutang0410@163.com (C.T.); xiuzhang11@163.com (X.L.); 13085500761@163.com (T.W.); 15574237597@163.com (M.X.); himi1228@163.com (M.H.); 2 State Key Laboratory for Conservation and Utilization of Bio-Resources in Yunnan, Yunnan University, Kunming 650091, China; wangjie1@stu.ynu.edu.cn; 3 Qinghai Institute of Health Sciences, Xining 810000, China; 15909715156@163.com

**Keywords:** *Cordyceps sinensis*, *Cordyceps militaris*, *Cordyceps cicadae*, untargeted metabolomics, differential metabolites

## Abstract

*Cordyceps* represent a valuable class of medicinal fungi with potential utilization. The overexploitation and resource scarcity of *Cordyceps sinensis* (CS) have led to the emergence of *Cordyceps* such as *Cordyceps militaris* (CM) and *Cordyceps cicadae* (CC) as substitutes. The medicinal value of CS is often considered superior to other *Cordyceps*, potentially owing to differences in active ingredients. This study aimed to evaluate the differences in the composition and abundance of the primary and secondary metabolites of CS and its substitutes by untargeted metabolomics. A total of 4671 metabolites from 18 superclasses were detected. CS and its substitutes were rich in amino acids, lipids, organic acids, and their derivatives. We statistically analyzed the metabolites and found a total of 285 differential metabolites (3′-Adenylic acid, O-Adipoylcarnitine, L-Dopachrome, etc.) between CS and CC, CS and CM, and CM and CC, which are potential biomarkers. L-glutamate and glycerophospholipids were differential metabolites. A KEGG enrichment analysis indicated that the tyrosine metabolic pathway and tryptophan metabolism pathway are the most differentially expressed pathways among the three *Cordyceps*. In contrast, CS was enriched in a higher abundance of most lipid metabolites when compared to CM and CC, which may be an indispensable foundation for the pharmacological functions of CS. In conclusion, systematic, untargeted metabolomics analyses for CS and other *Cordyceps* have delivered a precious resource for insights into metabolite landscapes and predicted potential components of disease therapeutics.

## 1. Introduction

*Cordyceps* is an entomopathogenic fungus that parasitizes larvae of moths [1]. It was one of the biggest genera in the Clavicipitaceae and was carved out in the ecological niche as an endoparasite of arthropods [2]. The distinctive stroma and sclerotia were the principal characteristics that distinguish *Cordyceps* from other fungi. Around 750 species of *Cordyceps* have been identified worldwide, with locations in South Asia, Europe, and North America. Still, only 35 species of *Cordyceps* have been reported for therapeutic use in folk medicine and have been identified based on their multiple bioactive ingredients [3]. Species of this genus are distinct from other common and high-yielding edible fungi, which are often expensive and extensively used as tonic and medicine for a variety of ailments [4], such as *Cordyceps militaris*, *Cordyceps sinensis*, *Cordyceps guangdongensis*, *Cordyceps gunnii*, and so on [4,5,6,7,8,9].

*C. sinensis*, the only species in the genus that is classified as a drug in the Chinese Pharmacopoeia, is a flashpoint for research on the genus *Cordyceps* [3]. It was widely distributed in the Qinghai–Tibet Plateau and its surrounding areas, containing the Qinghai, Tibet, Sichuan, and Yunnan provinces in China [9]. Its abundance of active ingredients, such as nucleosides, proteins, ergosterol, and polysaccharides, contributed to the increase in its significant economic value and used as a medicine in China for more than 300 years [10,11,12,13]. Recent investigations have shown its diverse pharmacological effects, encompassing hepatorenal protection, anti-inflammatory, hypoglycemic, and anticancer activities [14,15,16,17,18]. In recent years, *C. sinensis* production has declined due to climate change and overexploitation [19]. Therefore, finding suitable substitutes has become critical to ease the supply and demand of subhealth groups. Except for the fermentation of *Ophiocordyceps sinensis* and the cultivation of artificial Chinese cordyceps [20], an increasing number of people confirmed that other *Cordyceps* are expected to become substitutes for *C. sinensis*. *C. militaris* and *C. cicadae* also possess a similar effectiveness as *C. sinensis* [21]. *C. militaris*, also known as “YongChongCao”, has been used as a tonic for hundreds of years [6]. Previous pharmacological studies have confirmed that it is abundant in various active ingredients such as cordycepin and polysaccharides [22], which have significant therapeutic effects on a variety of diseases, containing antiproliferative, antimetastatic, and hypolipidemic properties [23]. *C. cicadae* is also exploited in traditional Chinese medicine (TCM), and contemporary research has proved that it has significant effects in the treatment of chronic renal failure, eye diseases, and dizziness [24,25]. Some studies have demonstrated that the nucleosides and some of the biological activities in *C. cicadae* were similar to those of *C. sinensis* [26]. Therefore, a comparative pharmacological and bioactive ingredients analysis suggests that *C. militaris* and *C. cicadae* are anticipated to be alternatives to *C. sinensis* in terms of medicinal value and health preservation.

*Cordyceps* normally infects a sole host or a few related species to evade the immunity of the host in seeking survival and propagation through a highly specific and sophisticated mechanism [27]. This specific approach is accompanied by the production of distinct secondary metabolites in response to host defenses [3]. Secondary metabolites of fungi were considered to be a rich source of natural products with potential medical applications [28]. For example, ganoderic acid β, a terpenoid with antiviral activity, and analogs of ganoderic acid were found in *Ganoderma lucidum* [29]. Lovastatin, an HMG-CoA reductase inhibitor widely found in oyster mushrooms, is used as a cholesterol-lowering agent, for the restoration of endothelial function, and for its anti-inflammatory activity [30]. Therefore, the study of metabolites of different *Cordyceps* is crucial for the discovery of new medicines and to explore the fundamental differences. Zhang et al. used widely targeted metabolomics to compare and analyze the metabolic differences between *C. sinensis* and *C. militaris*, *C. cicadae*, and *C. gunnii* and found that their metabolic profiles were more than 80% similar [31]. Nevertheless, a systematic and comprehensive approach to directly compare the metabolic differences in *C. sinensis* with those of *C. militaris* and *C. cicadae* is missing. In summary, to shed light on the key metabolic differences between *C. sinensis* and other *Cordyceps*, we used an untargeted metabolomics analysis with UHPLC-Q Exactive HF-X. Subsequently, we performed statistical and bioinformatic analyses to identify and contrast the differential metabolites between *C. sinensis* and other *Cordyceps*. Overall, our study explains the similarities and differences between *C. sinensis* and other *Cordyceps* in a complete set of primary and secondary metabolites, providing a theoretical basis for developing and utilizing *Cordyceps*.

## 2. Results

### 2.1. Metabolite Annotation and Comparative Analysis of Metabolite Profiles

**Figure 1:**
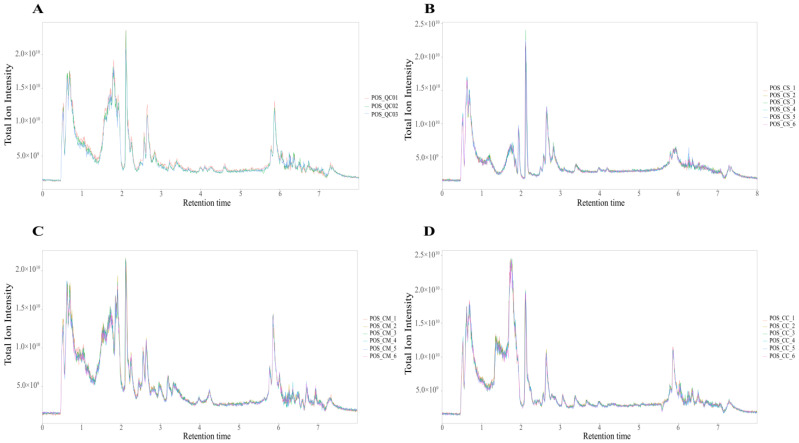
(**A**) Total ion chromatograms (TICs) of the QC samples. (**B**) TICs of the CS samples. (**C**) TICs of the CM samples. (**D**) TICs of the CC samples.

To obtain a clear distinction of metabolic profiling among *C. sinensis* (CS), *C. militaris* (CM)*,* and *C. cicadae* (CC), we characterized their primary and secondary metabolites by using UHPLC-Q Exactive HF-X untargeted metabolomics. The total ion chromatograms (TICs) of the QC, CS, CM, and CC samples are illustrated in Figure 1A, Figure 1B, Figure 1C, and Figure 1D, respectively. Biological replicates of the samples were analyzed, and the results showed that the retention times and peak intensities of the total ion chromatogram (TIC) overlapped, indicating that the MS maintained a stable signal when probing the same sample at different times.

**Figure 2:**
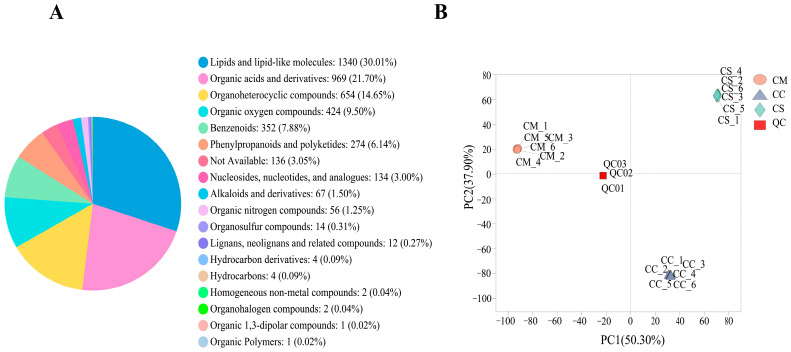
Metabolomics profiles of CS, CM, and CC. (**A**) Classification of the annotated metabolites in CS, CM, and CC. (**B**) Principal component analysis (PCA) results show metabolite profile differences between and within groups.

We annotated the metabolites based on multiple retrieval databases, and a total of 4671 metabolites were detected from 3 *Cordyceps*, including 2471 metabolites from ESI^+^ and 2200 metabolites from ESI^-^, and specific information is displayed in Tables S1 and S2. The detected metabolites were grouped into 18 superclasses according to the HMDB database shown in Figure 2A. Of all the metabolites, the most abundant superclass was lipids and lipid-like molecules, comprising 1340 metabolites (30.01%), followed by the organic acids and derivatives (969 metabolites, 21.70%), organoheterocyclic compounds (654 metabolites, 14.65%), and organic oxygen compounds (424 metabolites, 9.50%). A principal component analysis (PCA) of the samples contributes to visualizing the overall metabolic differentiation between groups and the degree of variability between samples within the same group. The overall PCA of all groups and QC samples showed that the metabolite profiles of the CS, CM, and CC were distinct (Figure 2B). The first main component explained 50.30% of the total variability while the second principal component accounted for 37.90% of the total variability of the data set, which indicated a clean separation of the samples by the components.

### 2.2. Screening of Differential Metabolites between C. sinensis and other Cordyceps

**Figure 3:**
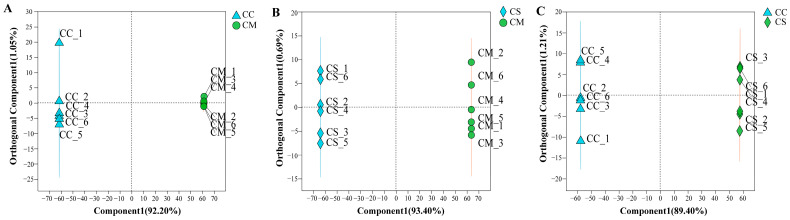
OPLS-DA score plots. (**A**) CC vs. CM. (**B**) CS vs. CM. (**C**) CC vs. CS. Comp1 first predicted principal component explanatory degree, orthogonal comp1 first predicted orthogonal component explanatory degree.

An orthogonal partial least squares discriminant analysis (OPLS-DA) is a multiple dependent to multiple independent variables regression-modeling method, which is effective at distinguishing pairwise comparison differences and improving the validity and resolution of the model [32]. To further demonstrate the differences between groups and identify the different metabolites, the metabolite contents were normalized and then subjected to the OPLS-DA model to obtain the OPLS-DA score plots. In the model, R^2^X (cum) and R^2^Y (cum) represent the interpretation rate of the built model to the X and Y matrix, respectively. Q^2^ indicates the forecasting ability of the model. The OPLS-DA results (Figure 3A–C) showed clear segregation between *C. sinensis* and other *Cordyceps*. We used the OPLS-DA model to compare the flavonoid metabolite composition of CC vs. CM (R^2^X = 0.922, R^2^Y = 1, Q^2^ = 1; Figure 3A), CS vs. CM (R^2^X = 0.934, R^2^Y = 1, Q^2^ = 1; Figure 3B), and CC vs. CS (R^2^X = 0.894, R^2^Y = 1, Q^2^ = 1; Figure 3C). High R^2^X, R^2^Y, and Q^2^ values were calculated for all pairwise comparisons, indicating that these analyses were reproducible, reliable, and suitable for screening differential metabolites.

**Figure 4:**
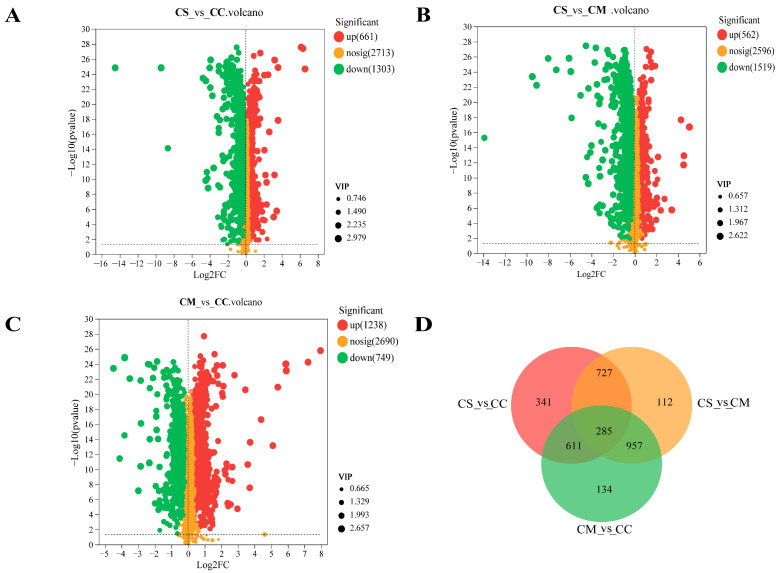
Differential metabolites of three species of *Cordyceps*. (**A**) Volcano plot of differential metabolites between CS vs. CC. (**B**) Volcano plot of differential metabolites between CS vs. CM. (**C**) Volcano plot of differential metabolites between CM vs. CC. (**D**) Venn plot of the number of differential metabolites among three comparison groups.

Volcano plots, as a category of scatter plots, are mainly used to show the differential metabolites of screening. In this study, differential metabolites screening was demonstrated based on the *p*-value < 0.05 and VIP > 1 criteria [33]. The screening results are represented as volcano plots (Figure 4A–C). There were 1964 (661 upregulated and 1303 downregulated) significantly differential metabolites between CS vs. CC, 2081 (562 upregulated and 1519 downregulated) between CS vs. CM, and 1987 (1238 upregulated and 749 downregulated) between CM vs. CC. The similarities and differences between *Cordyceps* were visually analyzed and a Venn diagram was generated (Figure 4D). We found that each *Cordyceps* has its own unique and differentiated metabolites, which demonstrate 341 differential metabolites in CC and 112 differential metabolites in CM. In addition, 285 common differential metabolites were observed, which were also potential biomarkers to distinguish three *Cordyceps*, and detailed information is displayed in Table S3. In general, of these common differential metabolites, we found that the three superclasses with the highest abundance were lipids and lipid-like molecules, organic acids and derivatives, and organoheterocyclic compounds, while organic acids and derivatives, organosulfur compounds, and alkaloids and derivatives were low in abundance.

### 2.3. Hierarchical Cluster Analysis Showed Distinctive Metabolite Profiles

**Figure 5:**
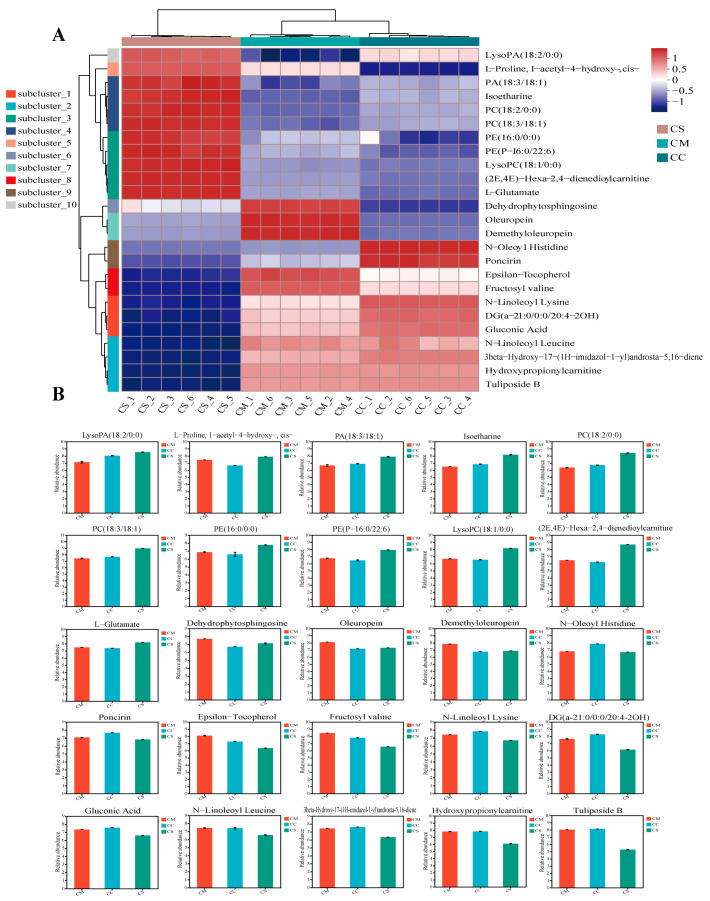
Hierarchical cluster analysis (HCA) revealed the variation in differential metabolites between and within groups. (**A**) Top 25 HCA of differential metabolites of 3 *Cordyceps*; (**B**) comparative analysis of top 25 differential metabolites in multiple groups. The horizontal coordinate indicates the group name and the vertical coordinate indicates the average relative abundance of metabolites in different groups.

The differential metabolites of the three *Cordyceps* were analyzed and evaluated by a hierarchical cluster analysis (HCA). Among the many differential metabolites, we visualized the top 25 differential metabolites and found that they were divided into 10 subclusters (Figure 5A), and the 10 subclusters are shown in Table S4. The results showed that many differential metabolites belonging to the superclass of lipids and lipid-like molecules were significantly overexpressed in CS. Compared with the other two groups of samples, the expression of lipid metabolites such as LysoPC(18:1/0:0), (2E,4E)-Hexa-2, 4-Dienedioylcarnitine, PA (18:3/18:1), Isoetharine, PC (18:2/0:0), and PA (18:3/18:1) was significantly higher than CC and CM (Figure 5B), while the oleuropein of the terpene glycosides and sesquiterpenoids belonging to terpene glycosides were significantly overexpressed in the CM samples. In addition, poncirin and N-oleoyl histidine in flavonoid glycosides, peptides, and analogs were found to have a significantly higher abundance in CC than in the other two samples. Notably, N-linoleoyl lysine, DG (a-21:0/0/20:4-2OH), and gluconic acid were significantly overexpressed in CC and CM. *N*-Linoleoyl Leucine; carbohydrates; carbohydrate conjugates of amino acids, peptides, and analogs; Tuliposide B; hydroxypropionylcarnitine; and 3-beta-Hydroxy-17-(1H-imidazol-1-yl) androsta-5,16-diene on androstane steroids have a high abundance in CM and CC. In summary, the differences in the metabolites of the three *Cordyceps* mainly exist in lipids and amino acids, which provides new insights for us to search for suitable biomarkers to distinguish.

### 2.4. Differential Metabolite Functional Pathway Analysis

**Figure 6:**
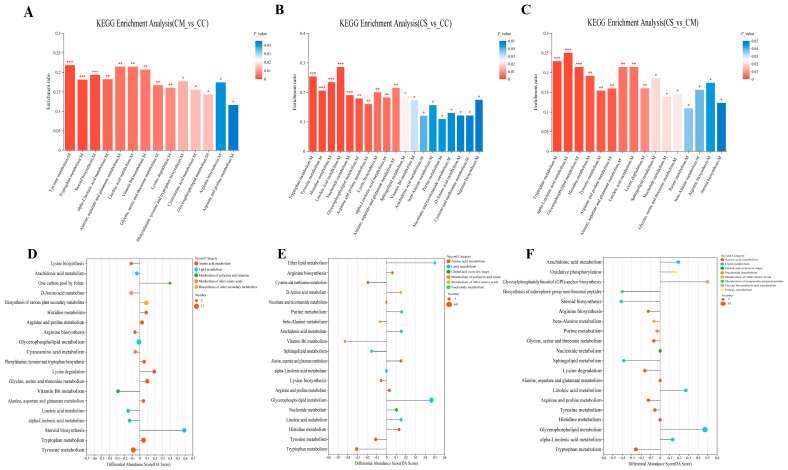
(**A**–**C**) KEGG enrichment analysis. The horizontal coordinate represents the pathway name, and the vertical coordinate represents the enrichment rate, which represents the ratio of the metabolite number enriched in this pathway to the background number annotated in the pathway. The larger the ratio, the greater the degree of enrichment. The column color gradient indicates the significance of enrichment. The darker the default color, the more significantly enriched the KEGG term, where the *p*-value or FDR < 0.001 is marked ***, the *p*-value or FDR < 0.01 is marked **, and the *p*-value or FDR < 0.05 is marked *. (**D**–**F**) KEGG pathway differential-abundance-score maps. The horizontal coordinate represents the DA score and the vertical coordinate represents the KEGG metabolic pathway name. DA score reflects the overall change in all metabolites in the metabolic pathway. A score of 1 indicates that the expression trend of all annotated differential metabolites in the pathway is upregulated; −1 indicates that the expression trend of all annotated differential metabolites in the pathway is downregulated; and the length of the line segment indicates the absolute value of DA score. The size of the dot indicates the number of differential metabolites annotated in the pathway, and the larger the dot, the more differential metabolites in the pathway. The dot distribution on the right side of the central axis and the longer line segment indicate that the overall expression of the pathway tends to be upregulated. The dots are distributed to the left of the central axis, and the longer the line segment, the more inclined the overall expression of the pathway is to downregulate. (**A**,**D**) CM vs. CC. (**B**,**E**) CS vs. CC. (**C**,**F**) CS vs. CM.

Based on the KEGG pathway database, we annotated and enriched the differential metabolites. The results showed that the differential metabolites in CM vs. CC were mainly significantly enriched in tyrosine metabolism, tryptophan metabolism, steroid biosynthesis and alpha-linolenic acid metabolism, and other pathways (Figure 6A). A differential score map analysis showed that the expression trend of differential metabolites in tyrosine metabolism and alpha-linolenic acid metabolism was downregulated. The differential metabolite expression of tryptophan metabolism and steroid biosynthesis were significantly upregulated (Figure 6D). In the CS vs. CC groups, differential metabolites were mainly enriched in tryptophan metabolism (downregulated), tyrosine metabolism (downregulated), histidine metabolism (upregulated), and linoleic acid metabolism (upregulated) (Figure 6B,E). In the CS vs. CM groups, differential metabolites were mainly enriched in tryptophan metabolism (downregulated), alpha-linolenic acid metabolism (upregulated), glycerophospholipid metabolism (upregulated), and histidine metabolism (Figure 6C,F).

In the intergroup comparison, we found that some metabolic pathways of the three *Cordyceps* overlapped and were significantly enriched, such as the tyrosine and tryptophan metabolic pathways. The differential metabolites in these two metabolic pathways may serve as potential biomarkers to distinguish the CS and other *Cordyceps*. In the tyrosine metabolic pathway, *N*-methyltyramine is highly expressed in CC and has the function of promoting appetite, digestion, and the absorption of nutrients [34]. 3,4-Dihydroxymandelic acid, an upregulated differential metabolite, is also significantly enriched and downregulated in CC and has a significant role in antioxidant and free-radical-scavenging activity [35]. However, the significantly upregulated differentially expressed metabolite of CM in the tryptophan metabolism pathway was 5-methoxytryptamine, which had the antioxidant effects of radiation protection and had more potential to develop CM into a new antioxidant compared with CC.

## 3. Discussion

Previous studies have demonstrated that the secondary products of TCMs are often crucial factors in determining their quality, which can directly affect their medicinal value [31]. The growth mechanism of *C. sinensis* is as complex as other *Cordyceps*, but due to the geographical location of growth, it has increased environmental stress compared with other *Cordyceps*. Therefore, there may be significant differences in the chemical composition and abundance of *Cordyceps*. A lack of studies comparing the complete metabolite profiles between *C. sinensis* and other *Cordyceps* with medicinal functions leads to difficulties in interpreting the differences in their metabolites and differences in their pharmacological functions. Here, we determined the complete metabolic profiles of different *Cordyceps* based on UHPLC-Q Exactive HF-X untargeted metabolomics and comparatively analyzed the metabolic profiles of three *Cordyceps*.

The Human Metabolic Database (HMDB, http://www.hmdb.ca) is a resource for metabolomic research and is the most comprehensive metabolic database, containing detailed information on the chemical structures, biological roles, and metabolic pathways of currently known human metabolites [36]. Furthermore, the HMDB database has a specialized database for food research, which contains food, microbial, and endogenous metabolite information, and is also valuable for TCM and food research [37]. Therefore, we annotated the natural metabolites in our samples with the purpose of providing a basis for clinical applications [38]. Herein, we detected 4671 metabolites in three *Cordyceps* based on multiple databases, most of which were metabolites from ESI^+^. According to the functional resemblance of the metabolites, we further categorized these metabolites into 18 superclasses. The most abundant superclasses are lipids and lipid-like molecules, organic acids and derivatives, organoheterocyclic compounds, organic oxygen compounds, benzenoids, etc. By screening the three *Cordyceps* by using multivariate statistical analysis, we found 1964 significantly different metabolites between CS vs. CC, 2081 different metabolites between CS vs. CM, and 1987 significantly different metabolites between CM vs. CC. This indicates that even within the same genus of entomopathogenic fungus, the types of metabolites are significantly different, which may be related to the host larvae and the growth environment. OPLS-DA also revealed that the metabolites contained in the three groups varied considerably. Following this, we performed an HCA analysis on differential metabolites and found that some superclasses of *C. sinensis* had a higher abundance of metabolites of lipids and lipid-like molecules such as PE (16:0/0:0), PE (P-16:0/22:6), LysoPC (18:1/0:0), (2E,4E)-Hexa-2,4-dienedioylcarnitine, etc., whereas organic acids and derivatives had less relative abundance. Interestingly, comparing the relative abundance values of the three *Cordyceps*, *C. sinensis* was enriched with more L-glutamate, which was in concordance with the results obtained by Guo et al. comparing wild and cultivated species of *Cordyceps* and mycelia [39]. L-glutamate serves as an essential substrate for protein synthesis that can be synthesized from other amino acids, and it plays an instrumental role in amino acid metabolism, with key functions in nutrition, metabolism, and signaling [40,41]. Therefore, differences in abundance due to possible differences in the underlying mechanisms of L-glutamate could be a potential marker for quality evaluation and metabolite annotation of the three species of *Cordyceps* [40]. In addition, since *Cordyceps* always had different flavors from other fungi, whether L-glutamate, as a fresh-flavored amino acid [42], has contributed to the flavors of different *Cordyceps* needs to be explored subsequently. Compared with CS and CM, CC is richer in Poncirin, a flavonoid glycoside derivative with prominent antitumor and antioxidant effects [43,44], which is the active differential metabolite that distinguishes the other two CS and CM.

In addition, we performed a KEGG pathway analysis to reveal differential pathways in all three *Cordyceps*. It is worth noting that the differential enrichment pathways between CS, CC, and CM depend on the synthesis of amino acids, lipids, and other bioactivities. In the second category, an intergroup comparison of the three *Cordyceps* revealed that the pathways were significantly enriched in amino acid metabolism and lipid metabolism. Moreover, the differential metabolites were significantly enriched in the tyrosine metabolic pathway and tryptophan metabolism. Of concern is the KEGG enrichment analysis of CS with CC and CM; we found 22 differential metabolites in tryptophan metabolism, including many secondary metabolites with active functions such as questiomycin that were significantly upregulated, and a metabolite with antimicrobial activity against gram-positive and gram-negative bacteria [45]. However, 5-methoxytryptamine, 5-hydroxykynurenine, and indolepyruvate were significantly reduced. In addition, we found a large proportion of lipid metabolism in the KEGG pathway differential-abundance scores of CS vs. CC and CS vs. CM, containing alpha-linolenic acid metabolism, glycerophospholipid metabolism, linoleic acid metabolism, and other pathways. Compared with CC and CM, CS has a higher relative abundance of various lipid metabolites. Lipids, as the basis of organism composition, are the mainstay of cell membranes, energy storage, and signaling molecules, which were found to be usually altered by ambient temperatures in previous studies [46]. Not merely that, fatty acid composition and cell membranes also strongly correspond to the environmental temperature. On the Tibetan Plateau, a region of extreme temperature variation, the survival of wild *C. sinensis* is accompanied by low-temperature stress [47], and the fungus usually adjusts its lipids by increasing unsaturated fatty acids [10]. A previous study found that wild *C. sinensis* has higher fatty acids, and this study is consistent with our study [48]. He et al. also compared *Isaria cicadae* with *C. sinensis* and found that significantly higher levels of sphingolipids were found in wild *C. sinensis* and artificially cultured *C. sinensis* insects but not in *I. cicadae*-cultured mycelium, which raised the possibility that immunostimulatory insects of *C. sinensis* can induce the fungus to produce more sphingolipids [49]. More lipid metabolites also have stronger immunosuppressive activity [50]; in our study, we found that CS is enriched with more glycerylphosphorylcholine, choline phosphate, and L-palmitoylphosphatidylcholine, which provides a potential basis for the development of the pharmacological function of CS and confirms that in regulating the immune function, CC and CM may be remarkably distinct from CS. Taken all together, after the metabolomics analysis on different *Cordyceps*, we found that their differential metabolites are significantly different, so *Cordyceps* development prospects may be distinguished in subsequent drug-development or clinical applications.

## 4. Materials and Methods

### 4.1. Materials and Reagents

**Figure 7:**
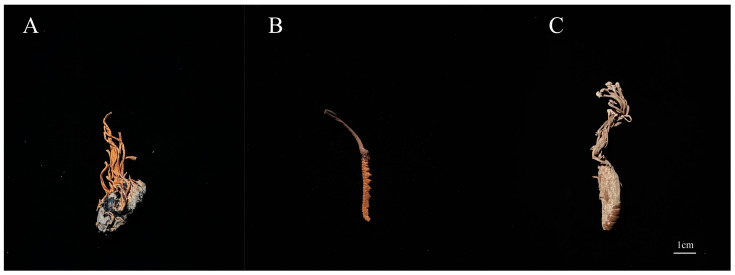
The specimens of mature *Cordyceps*. (**A**) *C. militaris*. (**B**) *C. sinensis*. (**C**) *C. cicadae*.

*C. militaris* (CM) and *C. cicadae* (CC) were purchased from Qinghai Baohuitang Biotechnology Co., Ltd. (Xining, China). *C. sinensis* (CS) was purchased from Qinghai Qingqitang Trading Co., Ltd. (Xining, China). Morphological details are shown in Figure 7.

### 4.2. Preparation of Sample Extracts

After the three groups of samples were cleaned separately with distilled water, the stroma and sclerotia of the samples were ground together into a solid powder. A 50 mg solid sample was added to a 2 mL centrifuge tube, and a 6 mm diameter grinding bead was added to 400 μL of extraction solution (methanol: water = 4:1 (*v:v*)) containing 0.02 mg/mL of internal standard (L-2-chlorophenylalanine), which was used for metabolite extraction. The samples were ground by the Wonbio-96c (Shanghai Wanbo Biotechnology Co., Ltd., Shanghai, China) frozen tissue grinder for 6 min (−10 °C, 50 Hz), followed by low-temperature ultrasonic extraction for 30 min (5 °C, 40 kHz). The samples were left at −20 °C for 30 min, centrifuged for 15 min (4 °C, 13,000× *g*), and the supernatant was transferred to the injection vial for LC-MS/MS analysis. Six biological replicates were used for each group.

### 4.3. Quality-Control Sample

As a part of the system conditioning and quality-control process, a pooled quality-control sample (QC) was prepared by mixing equal volumes of all samples. The QC samples were disposed of and tested in the same manner as the analytic samples. It helped to represent the whole sample set, which would be injected at regular intervals to monitor the stability of the analysis.

### 4.4. UHPLC-MS/MS Analysis

The LC-MS/MS analysis of the sample was conducted on a Thermo UHPLC-Q Exactive HF-X system equipped with an ACQUITY HSS T3 column (100 mm × 2.1 mm i.d., 1.8 μm; Waters, Milford, MA, USA) at Majorbio Bio-Pharm Technology Co., Ltd. (Shanghai, China). The mobile phases consisted of 0.1% formic acid in water: acetonitrile (95:5, *v*/*v*) (solvent A) and 0.1% formic acid in acetonitrile: isopropanol: water (47.5:47.5:5, *v*/*v*) (solvent B). Positive ion mode separation gradient: 0–3 min, mobile phase B was increased from 0% to 20%; 3–4.5 min, mobile phase B was increased from 20% to 35%; 4.5–5 min, mobile phase B was increased from 35% to 100%; 5–6.3 min, mobile phase B was maintained at 100%; 6.3–6.4 min, mobile phase B was decreased from 100% to 0%; and 6.4–8 min, mobile phase B was maintained at 0%. Separation gradient in negative ion mode: 0–1.5 min, mobile phase B rose from 0 to 5%; 1.5–2 min, mobile phase B rose from 5% to 10%; 2–4.5 min, mobile phase B rose from 10% to 30%; 4.5–5 min, mobile phase B rose from 30% to 100%; 5–6.3 min, mobile phase B linearly maintained 100%; 6.3–6.4 min, mobile phase B decreased from 100% to 0%; 6.4–8 min, mobile phase B was linearly maintained at 0%. The flow rate was 0.40 mL/min and the column temperature was 40 °C.

The mass spectrometric data were collected by using a UHPLC-Q Exactive HF-X Mass Spectrometer (Thermo Scientific, Waltham, MA, USA) equipped with an electrospray ionization (ESI) source operating in positive mode and negative mode. The optimal conditions were set as follows: source temperature at 425 °C; sheath gas flow rate at 50 arbs; Aux gas flow rate at 13 arbs; ion-spray voltage floating (ISVF) at −3500 V in negative mode and 3500 V in positive mode; and a stepped collision energy collection, normalizing collision energy, 20–40–60 rolling for MS/MS. The full MS resolution was 60,000 FWHM @ *m/z* 200, and the MS/MS resolution was 7500 FWHM @ *m/z* 200. Data acquisition was performed with the Data-Dependent Acquisition (DDA) mode, DDA collects the parent ion of top12, and FWHM sets 6 s. The detection was carried out over a mass range of 70–1050 *m*/*z.*

### 4.5. Data Analysis

The pretreatment of the LC/MS raw data was performed by using Progenesis QI version 3.0 (Waters Corporation, Milford, MA, USA) software, and a three-dimensional data matrix in CSV format was exported. The information in this three-dimensional matrix includes the sample information, metabolite name, and mass-spectral-response intensity. Internal standard peaks, as well as any known false-positive peaks (including noise, column bleed, and derivatized reagent peaks), were removed from the data matrix, made deredundant, and peak pooled. At the same time, the metabolites were annotated by searching databases [51], and the main databases were the HMDB (http://www.hmdb.ca/ (accessed on 27 July 2023)) [37]; Metlin (https://metlin.scripps.edu/ (accessed on 27 July 2023)); and the Majorbio Database, whose annotation level is level 2 and above [52]. Two levels of the Majorbio Database: metabolite annotation level B(i): accurate matching of Majorbio Database based on MS/MS secondary spectrum library; B(ii) accurate matching of MS/MS secondary spectrum library based on computer simulation. The raw LC-MS data were deposited in the MetaboLights with the accession number MTBLS8899.

The data matrix obtained by searching the database was uploaded to the Majorbio cloud platform (https://cloud.majorbio.com/ (accessed on 29 July 2023)) for data analysis. First, the data matrix was preprocessed as follows: At least 80% of the metabolic features detected in any set of samples were retained. After filtering, for specific samples with metabolite levels below the lower limit of quantification, the minimum metabolite value was estimated and each metabolic signature was normalized to the sum. To reduce the errors caused by sample preparation and instrument instability, the response intensities of the sample mass spectrometry peaks were normalized by using the sum normalization method to obtain the normalized data matrix. Meanwhile, the variables of the QC samples with a relative standard deviation (RSD) > 30% were excluded and log10 logarithmized to obtain the final data matrix for subsequent analysis.

Then, the R package “ropls” (Version 1.6.2) was used to perform a principal component analysis (PCA) and orthogonal least partial squares discriminant analysis (OPLS-DA) and 7-cycle interactive validation to evaluate the stability of the model. The metabolites with VIP > 1, *p* < 0.05 were determined to be significantly different metabolites based on the variable importance in the project (VIP) obtained by the OPLS-DA model and the *p*-value generated by the two-tailed Student’s *t*-test; an FDR correction was performed on the *p*-value.

Differential metabolites among the two groups were mapped into their biochemical pathways through a metabolic enrichment and pathway analysis based on the KEGG database (http://www.genome.jp/kegg/ (accessed on 2 August 2023)). These metabolites could be classified according to the pathways they are involved in or the functions they perform. An enrichment analysis was used to analyze whether a group of metabolites in a function node appears or not. The principle was that the annotation analysis of a single metabolite develops into an annotation analysis of a group of metabolites.

## 5. Conclusions

In conclusion, our current work depicts the metabolomic landscape of CS, CM, and CC based on UHPLC-Q Exactive HF-X untargeted metabolomics, and we found a comprehensive diversity of metabolites, including amino acids, and lipids were detected among the three *Cordyceps*. However, most of the biologically active lipids in CS relative abundance were higher than those in CM and CC, and interestingly, the relative abundance of L-glutamate was significantly different among the three *Cordyceps*. For CS, its hostile habitat and extremely variable environmental temperature resulted in significantly greater species and an abundance of most lipid metabolites compared with the other two species. Overall, this study provides a comprehensive understanding of the metabolites of CS, CM, and CC, which not only provides chemical evidence of the pharmacological functions of the three *Cordyceps* but also facilitates better quality evaluations.

## Data Availability

The data that support the findings of this study are available from the corresponding author upon reasonable request.

## Supplementary Materials

The following supporting information can be downloaded at https://www.mdpi.com/article/10.3390/molecules28237745/s1: Table S1: Detailed information of pos metabolites detected in CS, CM, and CC.; Table S2: Detailed information of neg metabolites detected in CS, CM, and CC.; Table S3: Common differential metabolites were detected in CS, CM, and CC.; Table S4: A total of 10 subcluster trends for CS, CM, and CC.
